# NSUN-Mediated m5C RNA Modification in Stem Cell Regulation

**DOI:** 10.3390/cells14201609

**Published:** 2025-10-16

**Authors:** Jiin Moon, Hyohi Lee, Yeonju Jang, Seung-Kyoon Kim

**Affiliations:** Department of Convergent Bioscience and Informatics, and Graduate School of Biological Sciences, Chungnam National University, Daejeon 34134, Republic of Korea

**Keywords:** RNA modification, 5-methylcytosine (m5C), NSUN, stem cells, pluripotency, differentiation

## Abstract

**Highlights:**

**What are the main findings?**

1. An integrated synthesis of m5C in stem cells centered on NSUNs, covering subcellular distribution, RNA substrates, and functional outputs.
2. A critical appraisal of the NSUN–m5C axis in cancer stem cells, including its potential clinical relevance.

**What is the implication of the main finding?**

3. A call for experiments to define bona fide m5C targets and causal mechanisms in stem cells and cancer stem cells.
4. A proposal of the NSUN-m5C pathway as a candidate diagnostic and prognostic biomarker and therapeutic target, and as evidence for testing its role as a cell state-specific RNA regulatory axis.

**Abstract:**

RNA modifications comprise a core epigenetic dimension of gene regulation; among these, N6-methyladenosine (m6A) and 5-methylcytosine (m5C) have been most intensively investigated. While the functions of m6A in stem cell biology have been well characterized, the contributions of m5C remain comparatively less well defined. This review focuses on m5C modifications catalyzed by the NSUN family of RNA methyltransferases and their roles in regulating stem cell identity, pluripotency, and differentiation. Evidence from embryonic and mesenchymal stem cells, as well as animal models, demonstrates that NSUN-mediated m5C is deposited on diverse RNA substrates, including rRNA, tRNA, mRNA, mitochondrial RNA, and enhancer RNAs, thereby influencing processes such as self-renewal, cell cycle progression, RNA stability, metabolic activation, and lineage specification. Disruption of m5C regulation often leads to developmental defects, underscoring its essential role during embryogenesis. Collectively, these findings establish m5C as a versatile and dynamic regulator in stem cell biology and underscore the need for future studies to delineate the roles of the NSUN family in stem cells and define the RNA targets of m5C. In addition, its broader implications for development, regenerative medicine, and disease, including cancer, as well as its potential interplay with other RNA modifications such as m6A and pseudouridine, remain important areas for further investigation.

## 1. Introduction

Among the various types of epigenetic regulation, RNA modifications constitute a diverse set of chemical marks that decorate RNA molecules, with more than 170 distinct modifications identified to date [1,2,3,4,5,6,7]. These include the well-studied N6-methyladenosine (m6A), 5-methylcytosine (m5C), pseudouridine (Ψ), N1-methyladenosine (m1A), and many others, whose biological functions are becoming increasingly clear [1,4,6]. RNA modifications occur across multiple RNA classes—mRNA, tRNA, rRNA, lncRNA, and snRNA—and their effects depend on the modification type, RNA substrate, cell type, and environmental context, highlighting the complex and dynamic roles of RNA modifications in gene regulation [1,4,7,8,9].

Stem cells are characterized by their capacity for self-renewal and pluripotency, which allow them to differentiate into multiple lineages in response to developmental and environmental cues [10,11,12]. Different types of stem cells, such as embryonic stem cells (ESCs), adult stem cells, and induced pluripotent stem cells (iPSCs), exhibit distinct differentiation potentials and efficiencies depending on their origins and developmental stages [13,14]. To sustain self-renewal while remaining poised for differentiation, stem cells rely on highly dynamic transcriptional programs that integrate diverse regulatory signals controlling both pluripotency and lineage specification [15,16].

Given these unique features, understanding the epigenetic regulation of stem cells is of fundamental importance. Although significant progress has been made in elucidating the role of m6A in stem cell biology [17,18,19], investigations into the functions of other RNA modifications remain relatively limited. In particular, m5C has only recently gained attention as a regulatory mark in mammalian cells. This modification is catalyzed mainly by members of the NOP2/Sun domain (NSUN) family of RNA methyltransferases, which includes enzymes such as NOP2, NSUN2, NSUN3, NSUN4, NSUN5, NSUN6, and NSUN7 [20,21]. Each family member exhibits distinct substrate specificities, targeting diverse classes of RNA, ranging from tRNAs and rRNAs to mRNAs and mitochondrial RNAs, thereby linking m5C to processes such as translation, RNA stability, and mitochondrial activity [22,23,24]. Among these enzymes, NSUN2 has been most extensively studied and is implicated in cell proliferation, differentiation, and stress responses [25,26,27,28,29,30], whereas the functions of other family members are only beginning to be explored.

In stem cell contexts, loss of proper m5C regulation has been shown to impair pluripotency, disrupt cell cycle progression, and cause developmental abnormalities, underscoring its essential role in embryogenesis [9,31,32]. However, compared with the well-characterized roles of m6A, our knowledge of m5C-mediated regulation remains fragmentary, and its downstream mechanisms and biological consequences in stem cells are not yet fully defined. Here, we synthesize current knowledge on NSUN-mediated m5C in stem cells and discuss its functional implications, with the aim of defining outstanding gaps and priorities for future work.

## 2. Molecular Mechanisms of NSUN-Mediated m5C RNA Modification

Similarly to other RNA modifications, m5C is governed by three classes of proteins: writers, which catalyze the addition of the modification; readers, which recognize and bind it to mediate downstream effects; and erasers, which remove the modification to restore the original cytosine state [6,8,33].

### 2.1. Writers

**Figure 1:**
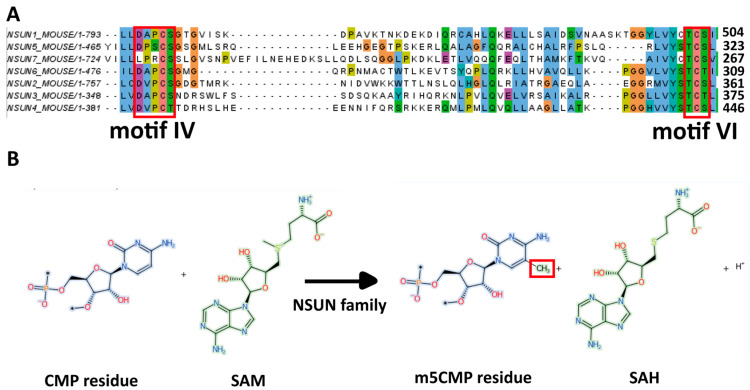
Conserved catalytic motifs in the NSUN family and the m5C methyl-transfer reaction. (**A**) Multiple sequence alignment of mouse NSUN1–NSUN7. Residues are colored by type using ClustalX coloring (Jalview; 2.11.5.0, Windows). Red boxes denote the conserved motif IV and motif VI. (**B**) Substrate state prior to catalysis: SAM (S-adenosyl-L-methionine) as the methyl donor and a target cytidine in RNA. Product state after NSUN-mediated transfer: formation of 5-methylcytidine (m5C) in RNA and release of SAH (S-adenosyl-L-homocysteine). The installed C5-methyl group is highlighted. Mouse NSUN sequences (UniProt, reviewed/canonical) were aligned with MAFFT and visualized in Jalview; motif boxes were added on alignment columns. Chemical depictions of Figure 1B were adapted from UniProt/ChEBI resources.

The deposition of m5C is primarily mediated by the NSUN family of RNA methyltransferases (NOP2/NSUN1-NSUN7) and by DNMT2, a DNA methyltransferase homolog [34,35,36,37,38]. This review focuses specifically on the NSUN family. Unlike DNMT2, which carries a single catalytic cysteine residue, NSUN proteins contain two catalytic cysteines in their active sites [39]. Additionally, the NSUN family installs m5C through the evolutionarily conserved motifs IV and VI [40] (Figure 1A). Mechanistically, NSUN enzymes use S-adenosylmethionine (SAM) as the methyl donor and convert it to S-adenosylhomocysteine (SAH) while methylating cytosine to form m5C [9,41] (Figure 1B).

#### 2.1.1. NOP2 (NSUN1)

Nucleolar protein 2 (NOP2), also known as NSUN1, is localized predominantly in the nucleolus and acts as a cytosine-C5 rRNA methyltransferase [42]. It is evolutionarily conserved across eukaryotes and modifies pre-rRNA to promote ribosome biogenesis. Beyond ribosomal RNA processing, NOP2 has also been linked to cell-cycle progression and proliferation [43,44].

#### 2.1.2. NSUN2

**Figure 2:**
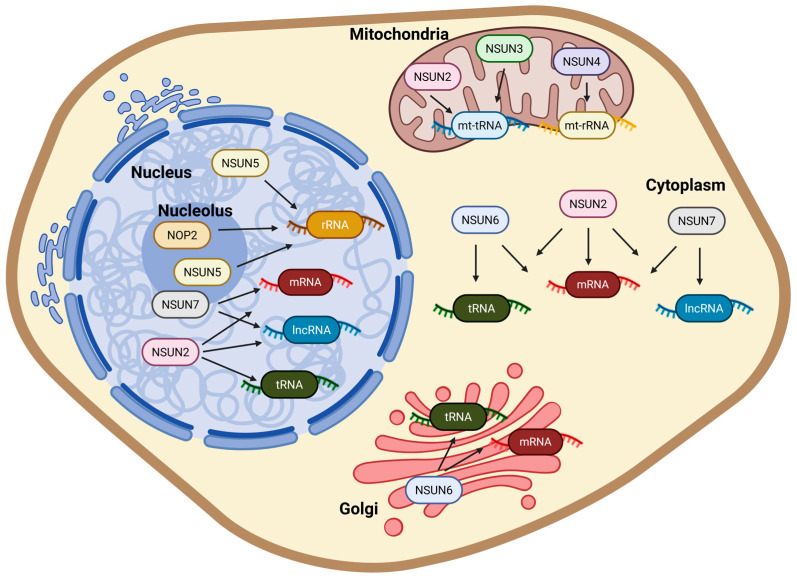
Cellular compartments and RNA substrates modified by NSUN family methyltransferases.

NSUN2 is the best-characterized member of the NSUN family. It is mainly localized in the nucleoplasm but can also be found in the cytoplasm during mitosis [31,45]. NSUN2 catalyzes m5C formation on diverse substrates, including mRNA, tRNA, mitochondrial tRNA (mt-tRNA), and specific long non-coding RNAs (lncRNAs) [9,46,47]. Functionally, NSUN2-catalyzed m5C enhances mRNA stability and translation efficiency [46]. In tRNAs, NSUN2 modifies cytosines at positions C34 and C48 of precursor tRNA-Leu (CAA), as well as C48-C50 of precursor tRNA-Gly (GCC), thereby influencing the generation of tRNA-derived fragments (tRFs) [9,48]. Moreover, NSUN2 also localizes to the mitochondrial matrix, where it catalyzes m5C on mitochondrial tRNAs, thereby linking its activity to mitochondrial function [47] (Figure 2).

#### 2.1.3. NSUN3

NSUN3 is restricted to the mitochondrial matrix and specifically methylates C34 (wobble position) of mt-tRNA-Met. This modification expands codon recognition, enabling the AUA codon to be decoded as methionine during mitochondrial translation [49] (Figure 2).

#### 2.1.4. NSUN4

Similarly to NSUN3, NSUN4 is another mitochondrial methyltransferase, targeting 12S mt-rRNA at C911 of the small ribosomal subunit. This modification is critical for mitochondrial ribosome assembly and function [9,50]. Recent studies indicate that NSUN4 can deposit m5C on mRNAs in a cell type-specific manner, with potential impacts on multiple cell-signaling pathways [51,52] (Figure 2).

#### 2.1.5. NSUN5

NSUN5 is localized in both the nucleolus and nucleoplasm. It methylates 28S rRNA in mammals and 25S rRNA in yeast, supporting ribosomal activity and protein synthesis [53,54]. Notably, the human *Nsun5* locus lies within a genomic region frequently deleted in Williams-Beuren syndrome, linking NSUN5 loss to multisystem developmental abnormalities [9,53,54] (Figure 2).

#### 2.1.6. NSUN6

NSUN6 primarily localizes to the cytoplasm and Golgi apparatus [55]. It selectively methylates C72 of tRNA-Cys and tRNA-Thr. NSUN6 also deposits m5C at CTCCA-containing stem-loop motifs within mRNA 3′UTRs, which stabilizes transcripts, fine-tunes translation output, and influences translation termination [55,56] (Figure 2).

#### 2.1.7. NSUN7

NSUN7 exhibits cell type-dependent subcellular localization, being detected in the cytoplasm, nucleolus, and nucleus, and has been reported to methylate both enhancer RNAs (eRNAs) and mRNAs [57,58,59], although its precise biological functions remain poorly understood. Enrichment in testis and a requirement for sperm flagellar development suggest tissue-specific functions, despite NSUN7 not being strictly essential for spermatogenesis itself [58] (Figure 2).

The NSUN family of RNA methyltransferases installs 5-methylcytosine (m5C) on diverse RNA species across distinct cellular compartments. NOP2/NSUN1 and NSUN5 target rRNA in the nucleolus and nucleoplasm. NSUN2 modifies mRNA, tRNA, and lncRNA in the nucleus and cytoplasm, and also mt-tRNA within mitochondria. NSUN3 and NSUN4 act in mitochondria on mt-tRNA and mt-rRNA, respectively. NSUN6 methylates specific tRNAs and mRNAs in the cytoplasm and Golgi apparatus. NSUN7 modifies mRNA and enhancer RNA (eRNA) in the cytoplasm, nucleolus, and nucleoplasm.

### 2.2. Readers

**Figure 3:**
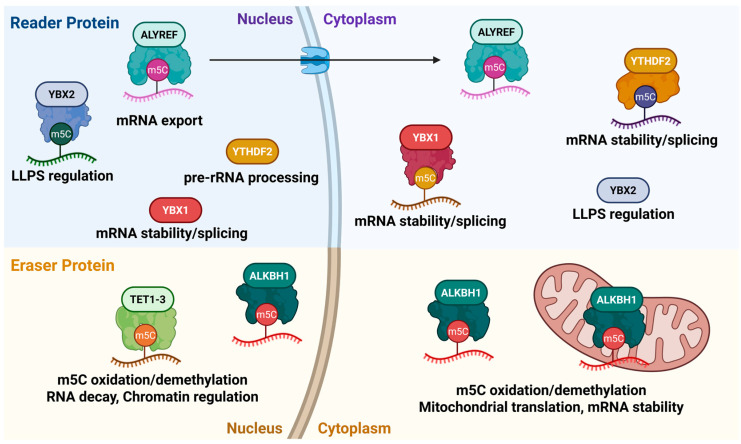
Reader and eraser proteins regulating m5C-modified RNA metabolism.

The biological consequences of m5C modifications are determined by reader proteins that selectively recognize and bind the modified nucleotide, thereby linking m5C to downstream processes such as RNA export, stability, splicing, and translation. Well-characterized m5C readers include ALYREF, YBX1, YBX2, and YTHDF2 [31] (Figure 3). Their distinct recognition modes emphasize that m5C is not a passive chemical mark but an active regulatory signal in RNA metabolism.

#### 2.2.1. ALYREF

Aly/REF export factor (ALYREF) functions as an mRNA export adaptor that shuttles between the nucleus and cytoplasm. By recognizing m5C modified transcripts, mediated in part by the comparatively conserved Lys-171 (K171) residue [34], ALYREF facilitates nuclear export and enhances mRNA stability [34,60,61]. Through these actions, ALYREF can modulate cellular programs, including those relevant to cancer [60].

#### 2.2.2. YBX1

Y-box binding protein 1 (YBX1) is present in both nucleus and cytoplasm and uses the indole ring of Trp-65 within its cold-shock domain to directly recognize m5C-modified mRNAs [25,62]. This interaction recruits additional RNA-binding proteins and regulates multiple post-transcriptional processes, including stability, splicing, and nuclear export [25,63,64].

#### 2.2.3. YBX2

Y-box binding protein 2 (YBX2), a member of the Y-box protein family, also interacts with m5C-modified mRNAs through the conserved Trp-100 within its cold shock domain (Trp-101 in mouse), and is present in both the nucleus and the cytoplasm [65]. Notably, YBX2 contributes to liquid–liquid phase separation (LLPS), thereby influencing RNA metabolism such as transcription, splicing, and translation [65,66].

#### 2.2.4. YTHDF2

Initially identified as an m6A reader [67,68], YTH N6-methyladenosine RNA-binding protein 2 (YTHDF2) has also been reported to recognize m5C in specific contexts [69,70]. Predominantly localized in the cytoplasm, YTHDF2 may interact with m5C through a conserved hydrophobic pocket centered on Trp-432 and has been observed to translocate to the nucleus under cellular stress [71]. It contributes to pre-rRNA processing, regulates mRNA stability, and participates in broader RNA metabolism [69,71]

#### 2.2.5. Other Readers

Beyond the best-characterized m5C readers-ALYREF, YBX1, YBX2, and YTHDF2-recent studies have identified additional proteins that may recognize m5C in specific contexts. FMRP preferentially binds m5C-modified RNA-DNA hybrids, thereby facilitating cellular m5C demethylation [72], and RAD52 has likewise been reported to engage m5C-bearing RNA-DNA hybrids more avidly in the setting of DNA damage-associated R-loops, linking m5C recognition to DNA repair pathways [73]. Moreover, the splicing factor SRSF2 has been shown to bind m5C and its expression or mutation status correlates with cancer prognosis [74]. As the repertoire of putative m5C-interacting proteins continues to expand, further systematic and orthogonal validation will be essential to define their binding specificity, structural determinants, and functional consequences in physiology and disease.

### 2.3. Erasers

The m5C mark deposited by the NSUN family is reversible. In mammals, its removal is mediated by dioxygenases such as TET1, TET2, and TET3 (Ten-eleven translocation family dioxygenases), which are primarily nuclear, and AlkB homolog 1 (ALKBH1), which is distributed across the nucleus, cytoplasm, and mitochondria [75,76,77]. These enzymes catalyze the stepwise oxidation of m5C to produce intermediates including hm5C (5-hydroxymethylcytosine) and f5C (5-formylcytosine) [72,78,79] (Figure 3). These enzymes are often referred to in the literature as m5C erasers in the operational sense that they remove the m5C state. Mechanistically, however, they oxidize m5C to hm5C or f5C. In particular, TET2 can generate hm5C, with reported roles in RNA decay, chromatin regulation, and transcriptional control [80,81]. ALKBH1 oxidizes m5C to hm5C and f5C, which has been implicated in mitochondrial translation and oxygen consumption as well as mRNA stability [82,83]. These observations suggest that hm5C and f5C may serve as functional RNA marks in their own right, rather than mere intermediates en route to full demethylation; further investigation is warranted to define their prevalence, mechanisms, and context-specific effects.

Reader proteins (top) recognize m5C-modified RNAs and regulate diverse aspects of RNA metabolism. ALYREF shuttles between the nucleus and cytoplasm to promote mRNA export. YBX1, present in both compartments, stabilizes mRNA and regulates splicing, while YBX2 contributes to liquid–liquid phase separation (LLPS). YTHDF2 is predominantly localized in the cytoplasm but can also function in the nucleus, where it has been implicated in pre-rRNA processing. Eraser proteins (bottom) catalyze the oxidation and removal of m5C. Nuclear TET1-3 and ALKBH1, which localizes to the nucleus, cytoplasm, and mitochondria, convert m5C into intermediates such as hm5C and f5C, thereby enabling dynamic regulation of RNA cytosine methylation.

### 2.4. Detection of m5C

To detect m5C in biological samples, researchers commonly use RNA bisulfite sequencing (RNA-BS-seq), MeRIP-m5C (antibody enrichment) sequencing, Aza-IP (5-azacytidine trapping), and miCLIP, as well as the more recent direct RNA sequencing (DRS) approach. Because each method differs in resolution, strengths, limitations, and practical caveats, selecting the appropriate assay according to the study’s objectives and experimental context is critical [36,84,85] (Table 1).

Side-by-side comparison of commonly used assays for mapping RNA m5C. The table summarizes resolution and key notes/cautions for RNA bisulfite sequencing (RNA-BS-seq), MeRIP-m5C, Aza-IP (5-azacytidine trapping), miCLIP, and direct RNA sequencing (DRS). Method-specific limitations-including incomplete bisulfite conversion in structured regions, antibody cross-reactivity and structure dependence, 5-azaC cytotoxicity and low-abundance detection sensitivity, input and workflow losses, and algorithm-dependent variability in DRS calls-are highlighted to explain why studies may report discordant sites or functions.

In summary, RNA m5C regulation results from the coordinated activities of NSUN writers, reader proteins that interpret the mark, and oxidative erasers that enable its reversibility. Comparatively, nucleolar/nuclear NSUN1/NSUN5 act on rRNA to support ribosome biogenesis; mitochondrial NSUN3/NSUN4 modify mt-tRNA and 12S mt-rRNA to sustain organellar translation; and NSUN2/NSUN6 regulate tRNA and selected mRNAs across multiple subcellular compartments. NSUN7 shows tissue-restricted, context-dependent activity-with reports of mRNA and eRNA methylation-suggesting specialized roles in testis (Figure 2). Readers such as ALYREF and YBX1/YBX2 route methylated transcripts toward nuclear export, RNP assembly/LLPS, or stabilized expression, whereas YTHDF2 links m5C to selective turnover in defined contexts. Oxidative enzymes underscore the reversibility of the mark and raise the possibility that hm5C and f5C function as regulatory states rather than mere intermediates (Figure 3). These assignments are supported by site-resolved mapping (RNA-BS-seq, MeRIP-m5C, Aza-IP, miCLIP, and direct RNA sequencing) together with genetic and functional readouts. At the same time, method-specific limitations materially shape interpretation: RNA-BS-seq can suffer from structure-dependent incomplete conversion; MeRIP-m5C from antibody cross-reactivity and structure dependence; Aza-IP from 5-azaC cytotoxicity and limited sensitivity to low-abundance targets; miCLIP from input/workflow losses and crosslinking bias; and DRS from signal-model and algorithmic variability (Table 1). Given these caveats-and the possibility that studies will report discordant sites or effects-cross-validation that combines multiple assays with genetics and function is essential. Conceptually, m5C represents a versatile and dynamic layer of post-transcriptional regulation, with broad implications for gene expression and cell fate, providing a reversible mechanism to fine-tune the transition from pluripotency to differentiation in a state- and context-dependent manner. This perspective is supported by genetic evidence across multiple systems: NSUN2-knockout mice display impaired tRNA methylation accompanied by defects in cell-cycle progression and synaptic differentiation [48,86], whereas loss of mitochondrial NSUN3 disrupts mt-tRNA modification and compromises oxidative phosphorylation [87]. Together, these genetic and functional studies form the foundation of our current understanding of NSUN diversity and its context-dependent activities.

## 3. Mechanistic Insights into m5C-Mediated Regulation of Stem Cell Fate

**Figure 4:**
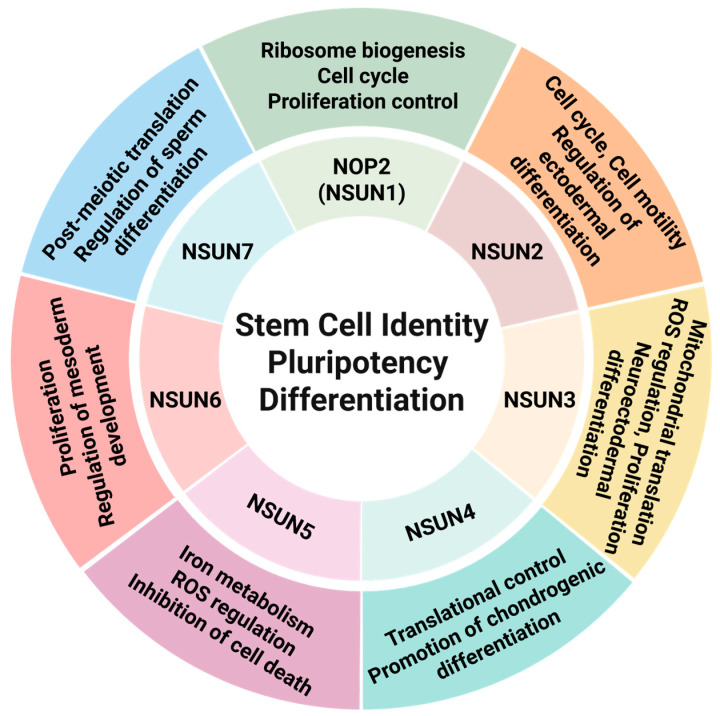
Functional roles of NSUN family members in stem cell biology.

The NSUN family of RNA methyltransferases is abundantly expressed in developing tissues and has been implicated in embryonic development [32]. In mouse embryonic stem cells (ESCs), global m5C levels are higher and more widely distributed than in brain tissues [88], suggesting greater diversity of deposition. In mesenchymal stem cells (MSCs), m5C is preferentially enriched within the first 100 nucleotides downstream of the translation initiation site [89], and ESCs display unique m5C sites whose patterns change during differentiation. Together, these observations indicate stage-specific programs of m5C deposition that operate during early embryogenesis and lineage commitment. Functionally, m5C modifications have been associated with cell cycle regulation, RNA processing and transport, chromatin modification, and developmental programs [37,90,91,92,93]. In this section, we outline how individual NSUN writer proteins shape stem cell identity and fate decisions (Figure 4).

NOP2 (NSUN1) is highly expressed in neural stem cells and declines during differentiation. It supports proliferative neural stem cell populations required for brain regeneration by modifying the 28S rRNA subunit, thereby promoting ribosome biogenesis, cell cycle progression, and stem cell proliferation [43,94].

NSUN2 is the most extensively studied NSUN enzyme. Loss of NSUN2 reduces tRNA methylation and drives the accumulation of tRNA-derived fragments, blunting neural stem-cell responses to growth factors such as FGF2 and resulting in impaired cell motility and aberrant neural development [48,95]. In skin stem cells, NSUN2 regulates tRNA-Leu methylation and c-Myc expression to maintain the balance between self-renewal and differentiation, with loss leading to cell cycle abnormalities and aberrant differentiation [86]. In ESCs, NSUN2 deposits m5C on mRNAs encoding pluripotency-related genes to stabilize transcripts, and its depletion compromises differentiation into neuroectodermal lineages [96].

NSUN3 is not strictly required for ESC pluripotency maintenance but supports proliferation and differentiation via mitochondrial translation. It also promotes proliferation by modulating Wnt signaling and mitochondrial reactive oxygen species (ROS). Loss of NSUN3 reduces mt-tRNA methylation and formylation, thereby impairing mitochondrial translation and respiration. Consequently, mitochondrial activation processes normally required during ESC differentiation, including increased mitochondrial mass and enhanced enzymatic activity, are disrupted. Functionally, NSUN3 deficiency suppresses neuroectodermal differentiation [49,87].

NSUN4 promotes chondrogenic differentiation in bone marrow-derived MSCs (BMSCs) by depositing m5C within the 3′UTR of *Sox9* mRNA. This activity cooperates with methyltransferase-like 3 (METTL3)-mediated m6A modification, leading to translational reprogramming that drives cartilage lineage commitment [97].

NSUN5 protects BMSCs from ferroptosis by regulating iron metabolism and ROS accumulation. Specifically, it modifies ferritin heavy chain 1 (FTH1) and ferritin light chain (FTL) transcripts to sustain protein synthesis, thereby antagonizing erastin-induced ferroptotic cell death and extending stem cell viability [98].

NSUN6 is required for proliferation and colony formation in BMSCs [99]. In human embryoid body differentiation models, NSUN6 depletion downregulates its target mRNAs and reduces the expression of mesodermal markers such as *Hoxa1*, implicating it in mesoderm and neural tube development [100].

NSUN7 is essential for spermatogenesis. Its loss impairs post-meiotic translation of mRNAs encoding proteins required for sperm function, leading to defects in germ cell differentiation [58,101,102,103].

Together, these findings indicate that m5C exerts diverse, context-dependent effects on stem cell identity, pluripotency, and lineage specification. Each NSUN family member acts on specific RNA substrates-including rRNA, tRNA, mRNA, and mitochondrial tRNA-to modulate translation, RNA stability, and cellular metabolism. These activities converge on key processes such as cell cycle progression, stress responses, and differentiation. While significant progress has been made in dissecting the functions of NSUN2 and NSUN3, the roles of other family members remain less understood. Future studies should aim to clarify their context-specific targets and interactions with other epigenetic modifications, which will be crucial for fully define how m5C contributes to stem-cell biology.

Schematic summary of NSUN family-mediated m5C functions in stem cells. Each NSUN protein contributes to distinct biological processes: NOP2/NSUN1 in ribosome biogenesis and cell cycle regulation; NSUN2 in pluripotency maintenance and ectodermal differentiation; NSUN3 in mitochondrial translation and reactive oxygen species (ROS) regulation; NSUN4 in chondrogenic differentiation; NSUN5 in iron metabolism and cell survival; NSUN6 in proliferation and mesoderm/neural tube development; and NSUN7 in post-meiotic translation and sperm differentiation. Collectively, these activities support stem cell identity, pluripotency, and lineage specification.

## 4. Roles of m5C RNA Modification in Embryonic Development

m5C is primarily associated with cell cycle regulation, RNA processing, transport, chromatin modification, and developmental pathways. In stem cells, NSUN family members broadly regulate both pluripotency and differentiation [104]. These stem cell-specific roles extend into embryogenesis, where m5C is crucial for lineage specification, germ layer formation, and overall embryo development [32] (Table 2).

NSUN2 is indispensable for early embryogenesis in *Drosophila*; by depositing m5C in mRNA 5′UTRs, it secures proper cell-cycle progression, and its loss delays development. Loss of NSUN2 delays embryonic development [105]. In mouse embryos, NSUN2 is broadly expressed during gastrulation (E7.5) and becomes enriched in neuroectoderm-derived tissues such as the cerebellum and forebrain as development proceeds [32,106].

NSUN3 is essential for mouse embryonic development, as knockout embryos are significantly smaller than wild-type and fail to survive gestation. Mechanistically, NSUN3 deficiency impairs mitochondrial respiration, disrupts cristae structure, and results in embryonic lethality. NSUN3 is highly expressed in the extraembryonic endoderm and lateral mesoderm at E7.5, although its precise developmental functions remain incompletely understood [107].

NSUN4 is also indispensable for embryogenesis. At E7.5, it is highly expressed in the outer embryo and neuroectoderm. Loss of NSUN4 causes structural defects in mt-rRNA, leading to impaired mitochondrial ribosome assembly and translation, which in turn produces embryonic lethality or severe developmental delays by E8.5 [50].

NSUN5 expression peaks around embryonic day 12 and decreases thereafter. NSUN5 knockout mice show cortical thinning during early development (postnatal day 10), particularly in layers II-V, accompanied by reduced dendritic arborization of layer V neurons. Mechanistically, NSUN5 deficiency suppresses CDC42 expression, impairing glial growth and migration. At E7.5, it is prominently expressed in mesoderm-derived tissues [108].

NSUN6 and NSUN7 are both highly expressed in embryos and extraembryonic tissues at E7.5. NSUN6 is enriched in developing neural tubes and somites, suggesting specific roles in neuroectodermal and axial development [32].

In addition to NSUN proteins, the RNA-binding protein Y-box binding protein 1 (YBX1), a known m5C reader, emerged as a critical regulator of mRNA stability during early zebrafish embryogenesis. Loss of YBX1 results in developmental arrest, underscoring its essential role in mRNA surveillance and stabilization during the earliest stages of life [63].

NSUN family proteins contribute to various aspects of embryonic development, including cell cycle regulation, mitochondrial function, ribosomal RNA modification, neurodevelopment, and germ cell maturation. The table summarizes their reported functions along with stage-specific expression patterns in mouse and *Drosophila* embryos.

Overall, these findings highlight the essential roles of m5C and its regulators during embryonic development. Each NSUN family member acts at specific developmental stages and tissues-from early gastrulation to neuroectodermal and mesodermal differentiation-by regulating RNA stability, translation, and mitochondrial function. Loss of these regulators commonly results in embryonic lethality or severe developmental abnormalities, highlighting the essential requirement of m5C for normal embryogenesis. Furthermore, the involvement of reader proteins such as YBX1 suggests that the functional impact of m5C extends beyond writers, reinforcing the complexity of this modification in shaping developmental trajectories.

## 5. m5C RNA Modification in Cancer Stem Cell Maintenance and Tumor Progression

Cancer stem cells (CSCs) share the fundamental properties of normal stem cells, such as self-renewal and multipotency, yet they constitute a distinct subpopulation capable of driving tumor progression. Beyond their self-renewal and multilineage potential, CSCs exhibit invasive traits essential for metastasis. Unlike normal stem cells, CSCs both produce heterogeneous progeny through unlimited divisions and display aneuploidy resulting from chromosomal rearrangements [109,110,111,112]. Telomere states in CSCs are heterogeneous. While some populations show shortening, many CSCs maintain telomeres through telomerase activation or alternative lengthening of telomeres (ALT), reflecting context-dependent dynamics [112,113,114,115]. Because CSCs express many of the same genes as normal stem cells, insights into stem cell biology provide a valuable framework for understanding CSC characteristics [109,110,111,112].

Among RNA modifications, m6A has been the most extensively studied and is established as a regulator of CSC functions [116,117]. Given that m6A is essential for embryonic development and supports CSC emergence and maintenance [117], the critical roles of m5C in embryogenesis imply that it may similarly influence CSC biology, warranting systematic investigation. For instance, NSUN7 is highly expressed in glioblastoma and correlates with poor prognosis. Mechanistically, NSUN7 deposits m5C on circular RNAs (circRNAs), stabilizing these transcripts, enhancing the expression of stemness markers, and sustaining self-renewal in glioblastoma stem-like cells [118]. In addition, the RNA demethylase TET2 functions in leukemia stem cells, where its depletion enhances self-renewal and migratory capacity, thereby accelerating leukemogenesis [119].

Beyond NSUN7, other NSUN family members have also been implicated in tumorigenesis [120]. Notably, NSUN2—the most extensively studied paralog—has been associated with the progression of hepatocellular carcinoma, pancreatic cancer, breast cancer, and other malignancies [121,122,123], NSUN5 has likewise been linked to hepatocellular carcinoma and gastric cancer [124,125], and NSUN6 has been reported in connection with cervical cancer and glioma [126,127]. While these observations highlight the oncogenic potential of NSUN proteins, their precise functions in CSC biology remain largely unexplored.

Taken together, these findings suggest that m5C modification contributes to the unique biology of CSCs by stabilizing stemness-related transcripts and modulating self-renewal and invasiveness. Although m6A is firmly established as a central regulator of CSC functions, emerging data from NSUN7 in glioblastoma and TET2 in leukemia highlight the potential significance of m5C in maintaining CSC properties and promoting tumor progression. Moreover, recent studies raise the possibility that m5C may functionally interact with other RNA modifications, including m6A and other RNA modifications, highlighting the potential significance of such cross-talk in regulating CSC plasticity [46,128,129]. Thus, investigating m5C dynamics in CSCs may provide new insights into the epigenetic regulation of malignancy and uncover novel therapeutic vulnerabilities.

## 6. Conclusions and Future Perspectives

m5C is a critical—but still incompletely understood—layer of epigenetic regulation across stem cells, embryonic development, and cancer stem cells. The NSUN family of “writers,” together with reader and eraser proteins, act on diverse RNA substrates, including rRNA, tRNA, mRNA, and enhancer RNAs, to regulate translation, RNA stability, cellular metabolism, and post-transcriptional processes. These regulatory activities converge on fundamental aspects of stem cell biology, such as self-renewal, pluripotency, lineage specification, and stress responses.

The conserved motifs IV and VI in the NSUN family underpin their catalytic activity and contribute to substrate recognition, influencing RNA stability and translation efficiency. Recent studies have begun to illuminate functional differences among NSUN proteins, reflecting variations in subcellular localization and RNA target specificity. Expanding the repertoire of high-throughput sequencing and experimental approaches beyond those described in Section 2.4 will be essential to fully elucidate the mechanistic diversity of NSUN-mediated RNA methylation and its broader implications for stem cell biology and developmental processes (Figure 1 and Figure 2, and Table 1).

At the stem cell level, NSUN2, NSUN3, and NSUN4 target distinct RNA substrates-tRNAs, mRNAs, and mitochondrial tRNAs, respectively, to regulate cell cycle progression, energy metabolism, and transcriptional programs. Among them, NSUN2 has emerged as a central regulator across neural, skin, and embryonic stem cells, maintaining the delicate balance between self-renewal and differentiation. Collectively, these findings establish m5C as a molecular mechanism that safeguards stem cell fate decisions, with implications for regenerative medicine and stem cell-based therapies.

During embryonic development, the loss of NSUN-mediated m5C frequently results in embryonic lethality or severe developmental abnormalities, underscoring its indispensable role in lineage commitment and tissue morphogenesis. In cancer stem cells, NSUN7 and TET2 have been implicated in sustaining self-renewal and invasiveness, highlighting how dysregulation of m5C contributes to tumor initiation and progression.

Despite these advances, significant gaps remain. The precise RNA targets of several NSUN proteins are still undefined, and recent studies suggest that m5C may functionally interact with other RNA modifications, including m6A [46,128,129], highlighting the potential significance of such cross-talk in stem cells—a concept that remains largely unexplored. In addition, the roles of reader proteins are only beginning to be characterized. Moreover, technical limitations, including incomplete bisulfite conversion, antibody cross-reactivity, and low stoichiometry of m5C sites, have contributed to inconsistent mapping results, further underscoring the fragmentary state of current knowledge. Future studies should aim to generate high-resolution maps of m5C across distinct stem cell states, clarify the molecular crosstalk among writers, readers, and erasers, and determine how aberrant m5C regulation contributes to disease pathogenesis.

Integrating m5C biology with stem cell and cancer research promises new biomarkers and therapeutic angles; defining how NSUN-mediated m5C programs gene expression and governs fate decisions could enable precision control of pluripotency, advance regenerative medicine, and selectively target cancer stem cells.

## Figures and Tables

**Table 1:** Comparative overview of RNA m5C detection methods.

Sequencing	Resolution	Notes and Cautions
RNA Bisulfite-seq (RNA-BS-seq)	Single-base resolution	Fails to deaminate base-paired/structured cytosines, leading to non-conversion and false positives.
		Bisulfite treatment induces substantial RNA degradation.
		Limited sensitivity at low input; cannot discriminate m5C from other cytosine modifications.
MeRIP-seq	Low resolution (~100–200 nt) Whereas deep sequencing enables high-resolution	Antibodies preferentially recognize single-stranded nucleic acids.
		RNA secondary structure can hinder detection of potential m5C sites.
Aza-IP	Single-base resolution	Labile or partially converted m5C sites may escape detection.
		5-azaC is cytotoxic to cells.
		Low-abundance RNAs may be poorly detected.
MiCLIP	Single-base resolution	Requires substantial input material.
		Multi-step RNA detection workflows reduce usable yield and negatively impact sensitivity.
Direct RNA Sequencing (DRS)	Single-base resolution	Results can vary across prediction algorithms.

**Table 2:** Expression patterns and functional roles of NSUN family proteins during embryogenesis.

Protein Name	Function/Expression	Embryonic Stage
NSUN2	Regulates cell cycle progression during early embryogenesis; essential for brain development	In *Drosophila* embryo
	Broadly expressed during gastrulation, including in ectoderm and forebrain (FB)	E7.5 in mouse embryos
NSUN3	Modulates mitochondrial respiratory complex activity, cristae organization, and mitochondrial size	E9.5 in mouse embryos
	Enriched in extraembryonic tissues, including allantois (AL), chorion (CH), and ectoderm (EE)	E7.5 in mouse embryos
NSUN4	Controls rRNA modification and mitochondrial ribosome biogenesis; regulates mitochondrial respiration and respiratory chain complex assembly	E7.5 in mouse embryos
	Highly expressed in the chorion (CH) and anterior neuroectoderm (ANE)	E8.5 in mouse embryos
NSUN5	Involved in brain development; maintains cortical thickness and laminar structure	E10 in mouse embryos
	Widely expressed in both embryonic and extraembryonic compartments	E7.5 in mouse embryos
NSUN6	Potentially involved in body axis elongation Exhibits widespread expression in embryonic and extraembryonic compartments, with modest enrichment in ANE	E7.5 in mouse embryos
NSUN7	Required for proper germ cell function Broadly expressed in embryonic and extraembryonic tissues, with slight enrichment in ANE	E7.5 in mouse embryos

## Data Availability

No new data were created or analyzed in this study.

## Abbreviations

The following abbreviations are used in this manuscript:

m6A	N6-methyladenosine
m5C	5-methylcytosine
Ψ (Psi)	Pseudouridine
m1A	N1-methyladenosine
mRNA	messenger RNA
tRNA	transfer RNA
rRNA	ribosomal RNA
lncRNA	long non-coding RNA
snRNA	small nuclear RNA
ESC	embryonic stem cell
iPSC	induced pluripotent stem cell
NSUN	NOP2/Sun RNA methyltransferase
SAM	S-adenosylmethionine
SAH	S-adenosylhomocysteine
ROS	Reactive oxygen species
BMSC	Bone marrow-derived mesenchymal stem cell
EE	Ectoderm
FB	forebrain
AL	Allantois
CH	Chorion
ANE	Anterior neuroectoderm
CSC	Cancer stem cell

## References

[1] Dunin-Horkawicz S., Czerwoniec A., Gajda M.J., Feder M., Grosjean H., Bujnicki J.M. (2006). MODOMICS: A database of RNA modification pathways. Nucleic Acids Res..

[2] Fu Y., Jia G., Pang X., Wang R.N., Wang X., Li C.J., Smemo S., Dai Q., Bailey K.A., Nobrega M.A. (2013). FTO-mediated formation of N 6-hydroxymethyladenosine and N 6-formyladenosine in mammalian RNA. Nat. Commun..

[3] Jia G., Fu Y., Zhao X., Dai Q., Zheng G., Yang Y., Yi C., Lindahl T., Pan T., Yang Y.-G. (2011). N 6-methyladenosine in nuclear RNA is a major substrate of the obesity-associated FTO. Nat. Chem. Biol..

[4] Meyer K.D., Saletore Y., Zumbo P., Elemento O., Mason C.E., Jaffrey S.R. (2012). Comprehensive analysis of mRNA methylation reveals enrichment in 3′ UTRs and near stop codons. Cell.

[5] Moore M.J. (2005). From birth to death: The complex lives of eukaryotic mRNAs. Science.

[6] Wiener D., Schwartz S. (2021). The epitranscriptome beyond m6A. Nat. Rev. Genet..

[7] Zheng G., Dahl J.A., Niu Y., Fu Y., Klungland A., Yang Y.-G., He C. (2013). Sprouts of RNA epigenetics: The discovery of mammalian RNA demethylases. RNA Biol..

[8] Khan M.A., Rafiq M.A., Noor A., Hussain S., Flores J.V., Rupp V., Vincent A.K., Malli R., Ali G., Khan F.S. (2012). Mutation in NSUN2, which encodes an RNA methyltransferase, causes autosomal-recessive intellectual disability. Am. J. Hum. Genet..

[9] Wang M.-K., Gao C.-C., Yang Y.-G. (2023). Emerging roles of RNA methylation in development. Acc. Chem. Res..

[10] Hess D.C., Borlongan C. (2008). Stem cells and neurological diseases. Cell Prolif..

[11] Jopling C., Boue S., Belmonte J.C.I. (2011). Dedifferentiation, transdifferentiation and reprogramming: Three routes to regeneration. Nat. Rev. Mol. Cell Biol..

[12] Orlacchio A., Bernardi G., Martino S. (2010). Stem cells: An overview of the current status of therapies for central and peripheral nervous system diseases. Curr. Med. Chem..

[13] Morena F., Armentano I., Montanucci P., Argentati C., Fortunati E., Montesano S., Bicchi I., Pescara T., Pennoni I., Mattioli S. (2017). Design of a nanocomposite substrate inducing adult stem cell assembly and progression toward an Epiblast-like or Primitive Endoderm-like phenotype via mechanotransduction. Biomaterials.

[14] Nichols J., Smith A. (2009). Naive and primed pluripotent states. Cell Stem Cell.

[15] Yu T., Zhang H., Zhang C., Ma G., Shen T., Luan Y., Zhang Z. (2025). CREB5 Promotes the Proliferation of Neural Stem/Progenitor Cells in the Rat Subventricular Zone via the Regulation of NFIX Expression. Cells.

[16] Weinberger L., Ayyash M., Novershtern N., Hanna J.H. (2016). Dynamic stem cell states: Naive to primed pluripotency in rodents and humans. Nat. Rev. Mol. Cell Biol..

[17] Batista P.J., Molinie B., Wang J., Qu K., Zhang J., Li L., Bouley D.M., Lujan E., Haddad B., Daneshvar K. (2014). m6A RNA modification controls cell fate transition in mammalian embryonic stem cells. Cell Stem Cell.

[18] Geula S., Moshitch-Moshkovitz S., Dominissini D., Mansour A.A., Kol N., Salmon-Divon M., Hershkovitz V., Peer E., Mor N., Manor Y.S. (2015). m6A mRNA methylation facilitates resolution of naïve pluripotency toward differentiation. Science.

[19] Wang Y., Li Y., Toth J.I., Petroski M.D., Zhang Z., Zhao J.C. (2014). N 6-methyladenosine modification destabilizes developmental regulators in embryonic stem cells. Nat. Cell Biol..

[20] Chen X., Yuan Y., Zhou F., Huang X., Li L., Pu J., Zeng Y., Jiang X. (2025). RNA m5C modification: From physiology to pathology and its biological significance. Front. Immunol..

[21] Chellamuthu A., Gray S.G. (2020). The RNA methyltransferase NSUN2 and its potential roles in cancer. Cells.

[22] Song H., Zhang J., Liu B., Xu J., Cai B., Yang H., Straube J., Yu X., Ma T. (2022). Biological roles of RNA m5C modification and its implications in Cancer immunotherapy. Biomark. Res..

[23] Reid R., Greene P.J., Santi D.V. (1999). Exposition of a family of RNA m 5 C methyltransferases from searching genomic and proteomic sequences. Nucleic Acids Res..

[24] Zhang T., Zhao F., Li J., Sun X., Zhang X., Wang H., Fan P., Lai L., Li Z., Sui T. (2024). Programmable RNA 5-methylcytosine (m5C) modification of cellular RNAs by dCasRx conjugated methyltransferase and demethylase. Nucleic Acids Res..

[25] Chen X., Li A., Sun B.-F., Yang Y., Han Y.-N., Yuan X., Chen R.-X., Wei W.-S., Liu Y., Gao C.-C. (2019). 5-methylcytosine promotes pathogenesis of bladder cancer through stabilizing mRNAs. Nat. Cell Biol..

[26] Gkatza N.A., Castro C., Harvey R.F., Heiß M., Popis M.C., Blanco S., Bornelöv S., Sajini A.A., Gleeson J.G., Griffin J.L. (2019). Cytosine-5 RNA methylation links protein synthesis to cell metabolism. PLoS Biol..

[27] Auxilien S., Guérineau V., Szweykowska-Kulińska Z., Golinelli-Pimpaneau B. (2012). The human tRNA m5C methyltransferase Misu is multisite-specific. RNA Biol..

[28] Brzezicha B., Schmidt M., Makałowska I., Jarmołowski A., Pieńkowska J., Szweykowska-Kulińska Z. (2006). Identification of human tRNA: m5C methyltransferase catalysing intron-dependent m5C formation in the first position of the anticodon of the. Nucleic Acids Res..

[29] Hussain S., Sajini A., Blanco S., Dietmann S., Lombard P., Sugimoto Y., Paramor M., Gleeson J., Odom D., Ule J. (2013). NSun2-mediated cytosine-5 methylation of vault noncoding RNA determines its processing into regulatory small RNAs. Cell Rep..

[30] Hussain S., Benavente S.B., Nascimento E., Dragoni I., Kurowski A., Gillich A., Humphreys P., Frye M. (2009). The nucleolar RNA methyltransferase Misu (NSun2) is required for mitotic spindle stability. J. Cell Biol..

[31] Zheng J., Lu Y., Lin Y., Si S., Guo B., Zhao X., Cui L. (2024). Epitranscriptomic modifications in mesenchymal stem cell differentiation: Advances, mechanistic insights, and beyond. Cell Death Differ..

[32] Chi L., Delgado-Olguín P. (2013). Expression of NOL1/NOP2/sun domain (Nsun) RNA methyltransferase family genes in early mouse embryogenesis. Gene Expr. Patterns.

[33] Shi H., Wei J., He C. (2019). Where, when, and how: Context-dependent functions of RNA methylation writers, readers, and erasers. Mol. Cell.

[34] Yang X., Yang Y., Sun B.-F., Chen Y.-S., Xu J.-W., Lai W.-Y., Li A., Wang X., Bhattarai D.P., Xiao W. (2017). 5-methylcytosine promotes mRNA export—NSUN2 as the methyltransferase and ALYREF as an m5C reader. Cell Res..

[35] Xue C., Chu Q., Zheng Q., Jiang S., Bao Z., Su Y., Lu J., Li L. (2022). Role of main RNA modifications in cancer: N6-methyladenosine, 5-methylcytosine, and pseudouridine. Signal Transduct. Target. Ther..

[36] Trixl L., Lusser A. (2019). The dynamic RNA modification 5-methylcytosine and its emerging role as an epitranscriptomic mark. Wiley Interdiscip. Rev. RNA.

[37] Nombela P., Miguel-López B., Blanco S. (2021). The role of m6A, m5C and Ψ RNA modifications in cancer: Novel therapeutic opportunities. Mol. Cancer.

[38] Motorin Y., Lyko F., Helm M. (2010). 5-methylcytosine in RNA: Detection, enzymatic formation and biological functions. Nucleic Acids Res..

[39] Moon H.J., Redman K.L. (2014). Trm4 and Nsun2 RNA: m5C methyltransferases form metabolite-dependent, covalent adducts with previously methylated RNA. Biochemistry.

[40] Bohnsack K.E., Höbartner C., Bohnsack M.T. (2019). Eukaryotic 5-methylcytosine (m5C) RNA methyltransferases: Mechanisms, cellular functions, and links to disease. Genes.

[41] Huber S.M., Van Delft P., Mendil L., Bachman M., Smollett K., Werner F., Miska E.A., Balasubramanian S. (2015). Formation and abundance of 5-hydroxymethylcytosine in RNA. Chembiochem.

[42] Liao H., Gaur A., McConie H., Shekar A., Wang K., Chang J.T., Breton G., Denicourt C. (2022). Human NOP2/NSUN1 regulates ribosome biogenesis through non-catalytic complex formation with box C/D snoRNPs. Nucleic Acids Res..

[43] Wu Q., Niebuhr E., Yang H., Hansen L. (2005). Determination of the ‘critical region’for cat-like cry of Cri-du-chat syndrome and analysis of candidate genes by quantitative PCR. Eur. J. Hum. Genet..

[44] Sloan K.E., Bohnsack M.T., Watkins N.J. (2013). The 5S RNP couples p53 homeostasis to ribosome biogenesis and nucleolar stress. Cell Rep..

[45] Zhang X., Liu Z., Yi J., Tang H., Xing J., Yu M., Tong T., Shang Y., Gorospe M., Wang W. (2012). The tRNA methyltransferase NSun2 stabilizes p16INK4 mRNA by methylating the 3′-untranslated region of p16. Nat. Commun..

[46] Li Q., Li X., Tang H., Jiang B., Dou Y., Gorospe M., Wang W. (2017). NSUN2-mediated m5C methylation and METTL3/METTL14-mediated m6A methylation cooperatively enhance p21 translation. J. Cell. Biochem..

[47] Shinoda S., Kitagawa S., Nakagawa S., Wei F.-Y., Tomizawa K., Araki K., Araki M., Suzuki T., Suzuki T. (2019). Mammalian NSUN2 introduces 5-methylcytidines into mitochondrial tRNAs. Nucleic Acids Res..

[48] Flores J.V., Cordero-Espinoza L., Oeztuerk-Winder F., Andersson-Rolf A., Selmi T., Blanco S., Tailor J., Dietmann S., Frye M. (2017). Cytosine-5 RNA methylation regulates neural stem cell differentiation and motility. Stem Cell Rep..

[49] Trixl L., Amort T., Wille A., Zinni M., Ebner S., Hechenberger C., Eichin F., Gabriel H., Schoberleitner I., Huang A. (2018). RNA cytosine methyltransferase Nsun3 regulates embryonic stem cell differentiation by promoting mitochondrial activity. Cell. Mol. Life Sci..

[50] Metodiev M.D., Spåhr H., Loguercio Polosa P., Meharg C., Becker C., Altmueller J., Habermann B., Larsson N.-G., Ruzzenente B. (2014). NSUN4 is a dual function mitochondrial protein required for both methylation of 12S rRNA and coordination of mitoribosomal assembly. PLoS Genet..

[51] Zhao Z., Zhou Y., Lv P., Zhou T., Liu H., Xie Y., Wu Z., Wang X., Zhao H., Zheng J. (2024). NSUN4 mediated RNA 5-methylcytosine promotes the malignant progression of glioma through improving the CDC42 mRNA stabilization. Cancer Lett..

[52] Cui M., Qu F., Wang L., Liu X., Yu J., Tang Z., Cheng D. (2022). m5C RNA methyltransferase-related gene NSUN4 stimulates malignant progression of hepatocellular carcinoma and can be a prognostic marker. Cancer Biomark..

[53] Janin M., Ortiz-Barahona V., de Moura M.C., Martínez-Cardús A., Llinàs-Arias P., Soler M., Nachmani D., Pelletier J., Schumann U., Calleja-Cervantes M.E. (2019). Epigenetic loss of RNA-methyltransferase NSUN5 in glioma targets ribosomes to drive a stress adaptive translational program. Acta Neuropathol..

[54] Heissenberger C., Liendl L., Nagelreiter F., Gonskikh Y., Yang G., Stelzer E.M., Krammer T.L., Micutkova L., Vogt S., Kreil D.P. (2019). Loss of the ribosomal RNA methyltransferase NSUN5 impairs global protein synthesis and normal growth. Nucleic Acids Res..

[55] Haag S., Warda A.S., Kretschmer J., Günnigmann M.A., Höbartner C., Bohnsack M.T. (2015). NSUN6 is a human RNA methyltransferase that catalyzes formation of m5C72 in specific tRNAs. RNA.

[56] Long T., Li J., Li H., Zhou M., Zhou X.-L., Liu R.-J., Wang E.-D. (2016). Sequence-specific and shape-selective RNA recognition by the human RNA 5-methylcytosine methyltransferase NSun6. J. Biol. Chem..

[57] Ortiz-Barahona V., Soler M., Davalos V., García-Prieto C.A., Janin M., Setien F., Fernández-Rebollo I., Bech-Serra J.J., De La Torre C., Guil S. (2023). Epigenetic inactivation of the 5-methylcytosine RNA methyltransferase NSUN7 is associated with clinical outcome and therapeutic vulnerability in liver cancer. Mol. Cancer.

[58] Guseva E.A., Averina O.A., Buev V.S., Bragina E.E., Permyakov O.A., Priymak A.V., Emelianova M.A., Romanov E.A., Grigoryeva O.O., Manskikh V.N. (2025). Testis-specific RNA methyltransferase NSUN7 contains a re-arranged catalytic site. Biochimie.

[59] Aguilo F., Li S., Balasubramaniyan N., Sancho A., Benko S., Zhang F., Vashisht A., Rengasamy M., Andino B., Chen C.-h. (2016). Deposition of 5-methylcytosine on enhancer RNAs enables the coactivator function of PGC-1α. Cell Rep..

[60] Nulali J., Zhang K., Long M., Wan Y., Liu Y., Zhang Q., Yang L., Hao J., Yang L., Song H. (2024). ALYREF-mediated RNA 5-methylcytosine modification promotes hepatocellular carcinoma progression via stabilizing EGFR mRNA and pSTAT3 activation. Int. J. Biol. Sci..

[61] Hu Y., Chen C., Tong X., Chen S., Hu X., Pan B., Sun X., Chen Z., Shi X., Hu Y. (2021). NSUN2 modified by SUMO-2/3 promotes gastric cancer progression and regulates mRNA m5C methylation. Cell Death Dis..

[62] Ray D., Kazan H., Chan E.T., Castillo L.P., Chaudhry S., Talukder S., Blencowe B.J., Morris Q., Hughes T.R. (2009). Rapid and systematic analysis of the RNA recognition specificities of RNA-binding proteins. Nat. Biotechnol..

[63] Yang Y., Wang L., Han X., Yang W.-L., Zhang M., Ma H.-L., Sun B.-F., Li A., Xia J., Chen J. (2019). RNA 5-methylcytosine facilitates the maternal-to-zygotic transition by preventing maternal mRNA decay. Mol. Cell.

[64] Capowski E.E., Esnault S.p., Bhattacharya S., Malter J.S. (2001). Y box-binding factor promotes eosinophil survival by stabilizing granulocyte-macrophage colony-stimulating factor mRNA. J. Immunol..

[65] Wang X., Wang M., Dai X., Han X., Zhou Y., Lai W., Zhang L., Yang Y., Chen Y., Wang H. (2022). RNA 5-methylcytosine regulates YBX2-dependent liquid-liquid phase separation. Fundam. Res..

[66] Sun H., Li K., Liu C., Yi C. (2023). Regulation and functions of non-m6A mRNA modifications. Nat. Rev. Mol. Cell Biol..

[67] Zaccara S., Jaffrey S.R. (2020). A unified model for the function of YTHDF proteins in regulating m6A-modified mRNA. Cell.

[68] Wang X., Lu Z., Gomez A., Hon G.C., Yue Y., Han D., Fu Y., Parisien M., Dai Q., Jia G. (2014). N 6-methyladenosine-dependent regulation of messenger RNA stability. Nature.

[69] Dai X., Gonzalez G., Li L., Li J., You C., Miao W., Hu J., Fu L., Zhao Y., Li R. (2019). YTHDF2 binds to 5-methylcytosine in RNA and modulates the maturation of ribosomal RNA. Anal. Chem..

[70] Chen Z., Zeng C., Yang L., Che Y., Chen M., Sau L., Wang B., Zhou K., Chen Y., Qing Y. (2025). YTHDF2 promotes ATP synthesis and immune evasion in B cell malignancies. Cell.

[71] Yu J., Li Y., Wang T., Zhong X. (2018). Modification of N6-methyladenosine RNA methylation on heat shock protein expression. PLoS ONE.

[72] Yang H., Wang Y., Xiang Y., Yadav T., Ouyang J., Phoon L., Zhu X., Shi Y., Zou L., Lan L. (2022). FMRP promotes transcription-coupled homologous recombination via facilitating TET1-mediated m5C RNA modification demethylation. Proc. Natl. Acad. Sci. USA.

[73] Chen H., Yang H., Zhu X., Yadav T., Ouyang J., Truesdell S.S., Tan J., Wang Y., Duan M., Wei L. (2020). m5C modification of mRNA serves a DNA damage code to promote homologous recombination. Nat. Commun..

[74] Ma H.-L., Bizet M., Da Costa C.S., Murisier F., de Bony E.J., Wang M.-K., Yoshimi A., Lin K.-T., Riching K.M., Wang X. (2023). SRSF2 plays an unexpected role as reader of m5C on mRNA, linking epitranscriptomics to cancer. Mol. Cell.

[75] Fu L., Guerrero C.R., Zhong N., Amato N.J., Liu Y., Liu S., Cai Q., Ji D., Jin S.-G., Niedernhofer L.J. (2014). Tet-mediated formation of 5-hydroxymethylcytosine in RNA. J. Am. Chem. Soc..

[76] Dominissini D., Nachtergaele S., Moshitch-Moshkovitz S., Peer E., Kol N., Ben-Haim M.S., Dai Q., Di Segni A., Salmon-Divon M., Clark W.C. (2016). The dynamic N 1-methyladenosine methylome in eukaryotic messenger RNA. Nature.

[77] Chen Y.S., Yang W.L., Zhao Y.L., Yang Y.G. (2021). Dynamic transcriptomic m5C and its regulatory role in RNA processing. Wiley Interdiscip. Rev. RNA.

[78] Shen H., Ontiveros R.J., Owens M.C., Liu M.Y., Ghanty U., Kohli R.M., Liu K.F. (2021). TET-mediated 5-methylcytosine oxidation in tRNA promotes translation. J. Biol. Chem..

[79] Wu X., Zhang Y. (2017). TET-mediated active DNA demethylation: Mechanism, function and beyond. Nat. Rev. Genet..

[80] Zou Z., Dou X., Li Y., Zhang Z., Wang J., Gao B., Xiao Y., Wang Y., Zhao L., Sun C. (2024). RNA m5C oxidation by TET2 regulates chromatin state and leukaemogenesis. Nature.

[81] Shen Q., Zhang Q., Shi Y., Shi Q., Jiang Y., Gu Y., Li Z., Li X., Zhao K., Wang C. (2018). Tet2 promotes pathogen infection-induced myelopoiesis through mRNA oxidation. Nature.

[82] Zhong J., Xu Z., Ding N., Wang Y., Chen W. (2024). The biological function of demethylase ALKBH1 and its role in human diseases. Heliyon.

[83] Zhang C., Li J., Wang L., Yang P., Luo X. (2025). ALKBH1 knockdown promotes the growth, migration and invasion of HTR-8/SVneo cells through regulating the m5C modification PSMD14. Sci. Rep..

[84] Xue C., Zhao Y., Li L. (2020). Advances in RNA cytosine-5 methylation: Detection, regulatory mechanisms, biological functions and links to cancer. Biomark. Res..

[85] Spangenberg J., Mündnich S., Busch A., Pastore S., Wierczeiko A., Goettsch W., Dietrich V., Pryszcz L.P., Cruciani S., Novoa E.M. (2025). The RMaP challenge of predicting RNA modifications by nanopore sequencing. Commun. Chem..

[86] Blanco S., Kurowski A., Nichols J., Watt F.M., Benitah S.A., Frye M. (2011). The RNA–methyltransferase Misu (NSun2) poises epidermal stem cells to differentiate. PLoS Genet..

[87] Haag S., Sloan K.E., Ranjan N., Warda A.S., Kretschmer J., Blessing C., Hübner B., Seikowski J., Dennerlein S., Rehling P. (2016). NSUN 3 and ABH 1 modify the wobble position of mt-t RNA Met to expand codon recognition in mitochondrial translation. EMBO J..

[88] Amort T., Rieder D., Wille A., Khokhlova-Cubberley D., Riml C., Trixl L., Jia X.-Y., Micura R., Lusser A. (2017). Distinct 5-methylcytosine profiles in poly (A) RNA from mouse embryonic stem cells and brain. Genome Biol..

[89] Roundtree I.A., Evans M.E., Pan T., He C. (2017). Dynamic RNA modifications in gene expression regulation. Cell.

[90] Morena F., Argentati C., Bazzucchi M., Emiliani C., Martino S. (2018). Above the epitranscriptome: RNA modifications and stem cell identity. Genes.

[91] Lu H., Xie Y., Tran L., Lan J., Yang Y., Murugan N.L., Wang R., Wang Y.J., Semenza G.L. (2020). Chemotherapy-induced S100A10 recruits KDM6A to facilitate OCT4-mediated breast cancer stemness. J. Clin. Investig..

[92] Gossage L., Murtaza M., Slatter A.F., Lichtenstein C.P., Warren A., Haynes B., Marass F., Roberts I., Shanahan S.J., Claas A. (2014). Clinical and pathological impact of VHL, PBRM1, BAP1, SETD2, KDM6A, and JARID1c in clear cell renal cell carcinoma. Genes Chromosomes Cancer.

[93] Squires J.E., Patel H.R., Nousch M., Sibbritt T., Humphreys D.T., Parker B.J., Suter C.M., Preiss T. (2012). Widespread occurrence of 5-methylcytosine in human coding and non-coding RNA. Nucleic Acids Res..

[94] Kosi N., Alić I., Kolačević M., Vrsaljko N., Milošević N.J., Sobol M., Philimonenko A., Hozak P., Gajović S., Pochet R. (2015). Nop2 is expressed during proliferation of neural stem cells and in adult mouse and human brain. Brain Res..

[95] Hsu Y.-C., Pasolli H.A., Fuchs E. (2011). Dynamics between stem cells, niche, and progeny in the hair follicle. Cell.

[96] Moon J., Lee H., Oh M., Jang Y., Um D., Kim T.-K., Kim S.-K. (2025). Embryonic stem cell-specific intragenic enhancer RNA essential for NSUN2-mediated stem cell fate regulation. Int. J. Biol. Macromol..

[97] Yang L., Ren Z., Yan S., Zhao L., Liu J., Zhao L., Li Z., Ye S., Liu A., Li X. (2022). Nsun4 and Mettl3 mediated translational reprogramming of Sox9 promotes BMSC chondrogenic differentiation. Commun. Biol..

[98] Liu J., Ren Z., Yang L., Zhu L., Li Y., Bie C., Liu H., Ji Y., Chen D., Zhu M. (2022). The NSUN5-FTH1/FTL pathway mediates ferroptosis in bone marrow-derived mesenchymal stem cells. Cell Death Discov..

[99] Hu S., Yang M., Xiao K., Yang Z., Cai L., Xie Y., Wang L., Wei R. (2023). Loss of NSUN6 inhibits osteosarcoma progression by downregulating EEF1A2 expression and activation of Akt/mTOR signaling pathway via m5C methylation. Exp. Ther. Med..

[100] Selmi T., Hussain S., Dietmann S., Heiß M., Borland K., Flad S., Carter J.-M., Dennison R., Huang Y.-L., Kellner S. (2021). Sequence-and structure-specific cytosine-5 mRNA methylation by NSUN6. Nucleic Acids Res..

[101] Frye M., Blanco S. (2016). Post-transcriptional modifications in development and stem cells. Development.

[102] Harris T., Marquez B., Suarez S., Schimenti J. (2007). Sperm motility defects and infertility in male mice with a mutation in Nsun7, a member of the Sun domain-containing family of putative RNA methyltransferases. Biol. Reprod..

[103] Khosronezhad N., Colagar A.H., Jorsarayi S.G.A. (2015). T26248G-transversion mutation in exon7 of the putative methyltransferase Nsun7 gene causes a change in protein folding associated with reduced sperm motility in asthenospermic men. Reprod. Fertil. Dev..

[104] Blanco S., Bandiera R., Popis M., Hussain S., Lombard P., Aleksic J., Sajini A., Tanna H., Cortés-Garrido R., Gkatza N. (2016). Stem cell function and stress response are controlled by protein synthesis. Nature.

[105] Liu J., Huang T., Chen W., Ding C., Zhao T., Zhao X., Cai B., Zhang Y., Li S., Zhang L. (2022). Developmental mRNA m5C landscape and regulatory innovations of massive m5C modification of maternal mRNAs in animals. Nat. Commun..

[106] Kim Y.A., Siddiqui T., Blaze J., Cosacak M.I., Winters T., Kumar A., Tein E., Sproul A.A., Teich A.F., Bartolini F. (2023). RNA methyltransferase NSun2 deficiency promotes neurodegeneration through epitranscriptomic regulation of tau phosphorylation. Acta Neuropathol..

[107] Murakami Y., Wei F.-Y., Kawamura Y., Horiguchi H., Kadomatsu T., Miyata K., Miura K., Oike Y., Ando Y., Ueda M. (2023). NSUN3-mediated mitochondrial tRNA 5-formylcytidine modification is essential for embryonic development and respiratory complexes in mice. Commun. Biol..

[108] Chen P., Zhang T., Yuan Z., Shen B., Chen L. (2019). Expression of the RNA methyltransferase Nsun5 is essential for developing cerebral cortex. Mol. Brain.

[109] Badve S., Nakshatri H. (2012). Breast-cancer stem cells—Beyond semantics. Lancet Oncol..

[110] Islam F., Qiao B., Smith R.A., Gopalan V., Lam A.K.-Y. (2015). Cancer stem cell: Fundamental experimental pathological concepts and updates. Exp. Mol. Pathol..

[111] Sampieri K., Fodde R. (2012). Cancer stem cells and metastasis. Semin. Cancer Biol..

[112] Manchanda A.S., Rai H.K., Kaur M., Arora P. (2024). Cancer stem cells targeted therapy: A changing concept in head and neck squamous cell carcinoma. J. Oral Maxillofac. Pathol..

[113] Silvestre D.C., Pineda J.R., Hoffschir F., Studler J.-M., Mouthon M.-A., Pflumio F., Junier M.-P., Chneiweiss H., Boussin F.D. (2011). Alternative lengthening of telomeres in human glioma stem cells. Stem Cells.

[114] Joseph I., Tressler R., Bassett E., Harley C., Buseman C.M., Pattamatta P., Wright W.E., Shay J.W., Go N.F. (2010). The telomerase inhibitor imetelstat depletes cancer stem cells in breast and pancreatic cancer cell lines. Cancer Res..

[115] Claude E., Decottignies A. (2020). Telomere maintenance mechanisms in cancer: Telomerase, ALT or lack thereof. Curr. Opin. Genet. Dev..

[116] Cerneckis J., Cui Q., Liu W., Shi Y. (2023). RNA modifications in cancer stem cell biology. Cancer Treat. Res..

[117] Zhang Z., Zhang C., Luo Y., Zhang G., Wu P., Sun N., He J. (2021). RNA N6-methyladenosine modification in the lethal teamwork of cancer stem cells and the tumor immune microenvironment: Current landscape and therapeutic potential. Clin. Transl. Med..

[118] Zhao Y., Liu X., Dong W., Lin H., Xu H., Yang J., Cui Z., Xue Y., Liu L., Wang P. (2022). NSUN7-Mediated m 5 c Modification of CircNTRK2 Regulates Stemness Properties of Glioblastoma Cells by Activating STK31. SSRNJ.

[119] Li Y., Xue M., Deng X., Dong L., Nguyen L.X.T., Ren L., Han L., Li C., Xue J., Zhao Z. (2023). TET2-mediated mRNA demethylation regulates leukemia stem cell homing and self-renewal. Cell Stem Cell.

[120] Qiu L., Jing Q., Li Y., Han J. (2023). RNA modification: Mechanisms and therapeutic targets. Mol. Biomed..

[121] Zhang X., An K., Ge X., Sun Y., Wei J., Ren W., Wang H., Wang Y., Du Y., He L. (2024). NSUN2/YBX1 promotes the progression of breast cancer by enhancing HGH1 mRNA stability through m5C methylation. Breast Cancer Res..

[122] Zhang G., Liu L., Li J., Chen Y., Wang Y., Zhang Y., Dong Z., Xue W., Sun R., Cui G. (2023). NSUN2 stimulates tumor progression via enhancing TIAM2 mRNA stability in pancreatic cancer. Cell Death Discov..

[123] Sun Z., Xue S., Zhang M., Xu H., Hu X., Chen S., Liu Y., Guo M., Cui H. (2020). Aberrant NSUN2-mediated m5C modification of H19 lncRNA is associated with poor differentiation of hepatocellular carcinoma. Oncogene.

[124] Liu S., Liu Y., Zhou Y., Xia G., Liu H., Zeng Y., Pei Z., Cao J., Jing G., Zou H. (2024). NSUN5 promotes tumorigenic phenotypes through the WNT signaling pathway and immunosuppression of CD8+ T cells in gastric cancer. Cell. Signal..

[125] Gu X., Li P., Gao X., Ru Y., Xue C., Zhang S., Liu Y., Hu X. (2024). RNA 5-methylcytosine writer NSUN5 promotes hepatocellular carcinoma cell proliferation via a ZBED3-dependent mechanism. Oncogene.

[126] Yu M., Ni M., Xu F., Liu C., Chen L., Li J., Xia S., Diao Y., Chen J., Zhu J. (2024). NSUN6-mediated 5-methylcytosine modification of NDRG1 mRNA promotes radioresistance in cervical cancer. Mol. Cancer.

[127] Awah C.U., Winter J., Mazdoom C.M., Ogunwobi O.O. (2021). NSUN6, an RNA methyltransferase of 5-mC controls glioblastoma response to temozolomide (TMZ) via NELFB and RPS6KB2 interaction. Cancer Biol. Ther..

[128] Abbasi-Moheb L., Mertel S., Gonsior M., Nouri-Vahid L., Kahrizi K., Cirak S., Wieczorek D., Motazacker M.M., Esmaeeli-Nieh S., Cremer K. (2012). Mutations in NSUN2 cause autosomal-recessive intellectual disability. Am. J. Hum. Genet..

[129] Boo S.H., Ha H., Kim Y.K. (2022). m1A and m6A modifications function cooperatively to facilitate rapid mRNA degradation. Cell Rep..

